# Quality of care is key to tackling Mexico’s diabetes emergency

**DOI:** 10.2471/BLT.17.020617

**Published:** 2017-06-01

## Abstract

Mexico has declared the epidemic of diabetes a national emergency and is seeking to improve the quality of care for some 13 million people with the disease. Amy Guthrie and Fiona Fleck report.

In a clinic in Mexico City’s Roma Norte neighbourhood a doctor calls the 12 patients by name. One by one they are weighed, their blood pressure is taken and waist circumference measured.

At these monthly sessions run by the DiabetIMSS programme, patients learn how to manage type 2 diabetes – a chronic condition that is not immediately life-threatening as long as they take their medicine and make lifestyle changes. 

“The patients learn to keep their disease in check to avoid serious complications like amputations,” explains Dr Sara Leticia Arana Barriga, head of the clinic’s DiabetIMSS programme, run by the Instituto Mexicano del Seguro Social (IMSS), which is part of Mexico’s national public health system.

While the onus is partly on patients to adhere to treatment and diet advice to stay well and ward off life-threatening complications, health-care services must also ensure that people – once diagnosed with diabetes – have access to medicines, are regularly screened for complications, and that any complications are treated promptly.

Type 2 diabetes is the leading cause of death and disability combined in Mexico, according to the Global Burden of Disease project. Last year the Mexican health ministry declared the diabetes epidemic a national health emergency.

Under the National Strategy for the Prevention and Control of Overweight, Obesity and Diabetes launched in 2013, Mexico has been fighting the epidemic on three fronts: public health, medical care, and fiscal and regulatory policies.

The country has taken steps to improve its first line of defence: prevention, through its soda tax and public awareness campaigns. Boosting detection of diabetes at primary care level to find cases earlier on and to provide treatment and care combined with education is done to help extend the lives of those diagnosed with the disease.

For Mexico’s Undersecretary of Prevention and Health Promotion, Dr Pablo Kuri-Morales, tracking progress towards the goals of the national strategy is essential for improving the quality of care for people with chronic conditions.

“We are tracking progress through the Mexican Observatory on Noncommunicable Diseases,” Kuri-Morales says, referring to an independent monitoring agency run by the Autonomous University of Nuevo Leon launched in 2015.

The observatory has several tools designed to track progress, including both the Chronic Disease Information System and the Diabetes Patient Care Quality Index developed by the Carlos Slim Foundation.

“Data on patients are gathered by the primary care clinics using standardized protocols. These data are entered in the Chronic Disease Information System and processed by the Diabetes Patient Care Quality Index to compare the health-care outcomes in more than 12 000 primary health-care clinics in the districts, states and at the national level,” explains Dr Roberto Tapia-Conyer, Chief Executive Officer of the Carlos Slim Foundation. 

Mexico accounts for the most hospitalizations related to diabetes among the Organisation for Economic Co-operation and Development’s (OECD) 35 countries. 

But now deficiencies in diabetes care, such as the failure to detect complications in diabetes patients, become apparent via the Diabetes Patient Care Quality Index so that corrective action can be taken in the clinics or districts concerned, and many of these hospitalizations can be avoided, says Tapia-Conyer. 

For Eduardo Gonzalez-Pier, who held senior positions for more than 20 years in the health and social security sectors in Mexico, these are promising initiatives to improve the quality of care and – ultimately – health outcomes in Mexico.

“Quality of care is a big challenge in Mexico, and the world round,” says Gonzalez-Pier, a distinguished visiting fellow at the Center for Global Development in Washington, DC, United States of America.

“Diabetes is a particularly complicated case: you do many things and there are still no significant results,” Gonzalez-Pier says.

A 2016 OECD review of the Mexican health system highlighted quality of care as a major concern and called for the establishment of a new regulatory agency to assure the quality of health care.

Mexico is not alone. The *Lancet Global Health* journal launched a commission in March bringing together 30 policy-makers and health experts from 18 countries to consider ways in which low- and middle-income countries can improve health-care quality.

Improving the quality of care is vital for addressing Mexico’s type 2 diabetes epidemic – a task that is fraught with challenges.

An estimated 13 million people aged over 20 years of age in this Latin American country of 121.5 million people are living with diabetes, according to Mexico’s 2016 mid-term National Health and Nutrition Survey, but only 50–60% of them have been formally diagnosed.

“One of the big quality problems with diabetes care is timely detection and this can be seen across the whole of Latin America. In many of these health systems, patients arrive late, when their condition has reached an advanced stage,” Gonzalez-Pier says.

“One of the big quality problems with diabetes care is timely detection and this can be seen across the whole of Latin America.”

Eduardo Gonzalez-Pier

“Thirty years ago – when the main causes of death were infectious diseases – the health systems mainly attended to acute problems: respiratory and intestinal infections, and births and accidents. When the issues are acute – fever, diarrhoea or bleeding – the population comes forward.

“Now the main causes of death are noncommunicable diseases, such as heart disease, stroke and diabetes, but the system hasn’t transformed into a proactive system that encourages people to come forward regularly for check-ups,” he says.

Timely detection of diabetes and other conditions is an important component of a recent Ministry of Health initiative called CASALUD that uses new technology and other approaches to deliver noncommunicable disease prevention, screening, care and control. 

“Health professionals screen individuals using the Integrated Measurement for Early Detection approach that classifies them as healthy or in a pre-disease state – pre-obesity, pre-diabetes and pre-hypertension – using specially designed software for this,” explains Tapia-Conyer, who helped to develop the approach at the Carlos Slim Foundation. More than 600 000 individuals have been screened in this way, he says. 

Many Mexicans are diagnosed with diabetes at a relatively early age with 3.25% of cases detected between 20 and 39 years, compared to an OECD average of 1.7%, according to the recent review.

This is worrying, because early onset increases the risk of serious complications and this has repercussions in terms of premature disability and mortality, as well as cost – all of which is unnecessary because diabetes is a preventable disease.

“We need to re-conceptualize the way that we address health problems because our main challenge is chronic diseases,” says Dr Rafael Lozano, director of the Centre for Health Systems Research at Mexico’s National Institute of Public Health.

“We need to re-conceptualize the way that we address health problems because our main challenge is chronic diseases.”

Rafael Lozano

Lozano says that the mindset of the typical Mexican health-care provider is still lodged in the 20th century: they are prepared to deal with infections or acute episodes, but do not think in terms of the need for follow-up and management of chronic disease over time.

Since 2004, Mexico has extended health-care coverage to 52 million previously unenrolled Mexicans through the Seguro Popular programme. But this recent achievement of universal coverage of health services has not translated into high quality of care for all.

In Mexico, the incentive is to provide services to a large number of patients, rather than to achieve good health outcomes, Lozano says.

The private sector is slightly more proactive, but when it comes to chronic diseases, patients often rely on public networks such as IMSS due to the high cost of treatment and care. IMSS says that diabetes is the most costly illness for its network, and is the leading cause of blindness, amputations and chronic renal failure for its patients.

Mexicans are living longer. World Bank data show that the average Mexican can expect to reach 77 years compared with 57 in 1960. But public health clinics and hospitals are understaffed, medications can be scarce and the country’s fragmented health system does not help matters.

IMSS provides health-care coverage for private sector employees, as well as pensioners and their families. The Instituto de Seguridad y Servicios Sociales de los Trabajadores del Estado serves government employees and their families. The Seguro Popular provides care to the poorest Mexicans, while affluent Mexicans often opt for private health care in addition to public schemes. 

Many Mexicans are covered by more than one health-care insurance plan.

While Mexico has rolled out universal health coverage in principle, Mexicans contribute substantial out-of-pocket payments towards their health-care costs accounting for some 45% of total health spending, according to the OECD review, and some Mexicans do not realize they are covered by health insurance and, therefore, do not benefit from it.

The rise of type 2 diabetes in Mexico has laid bare this fragmentation and fundamental weakness in the health system.

“Mexico is facing a major challenge. The epidemics of diabetes, overweight and obesity and the consequences of these chronic conditions can only be dealt with through a comprehensive and integrated approach,” Kuri-Morales says.

**Figure:**
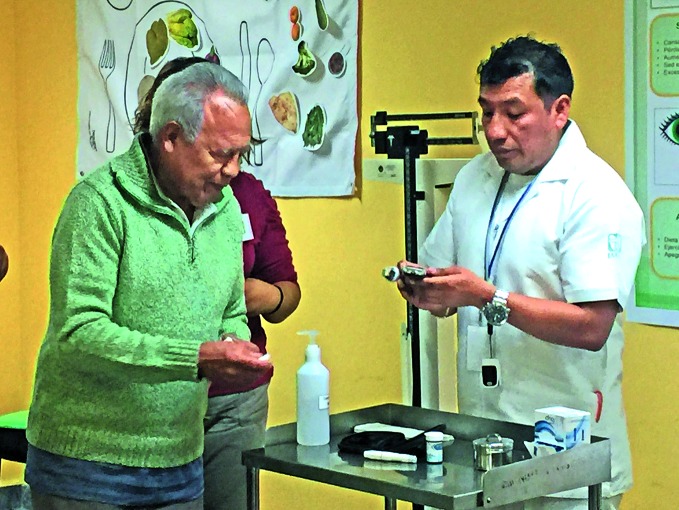
Diabetes patient Silvestre Sánchez has his blood glucose read by a nurse at the Roma Norte neighbourhood health clinic in Mexico City.

Amy Guthrie

**Figure:**
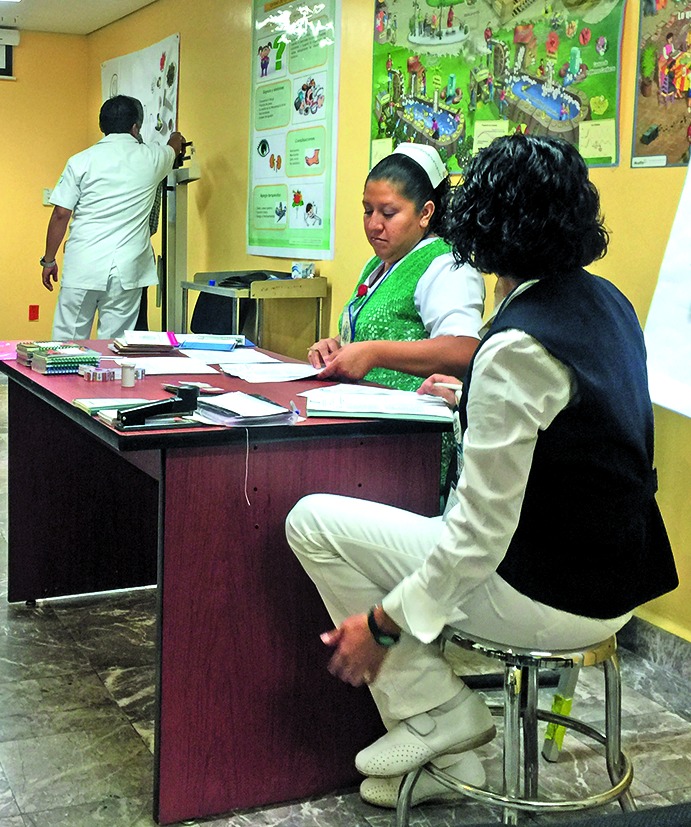
A DiabetIMSS session at the Roma Norte neighbourhood health clinic in Mexico City.

Amy Guthrie

